# Enhancing Lesion Segmentation in Ultrasound Images: The Impact of Targeted Data Augmentation Strategies

**DOI:** 10.1155/ijbi/3309822

**Published:** 2025-08-11

**Authors:** Xu Wang, Patrice Monkam, Bonan Zhao, Shouliang Qi, He Ma, Long Huang, Wei Qian

**Affiliations:** ^1^College of Medicine and Biological Information Engineering, Northeastern University, Shenyang, Liaoning, China; ^2^Key Laboratory of Intelligent Computing in Medical Image, Ministry of Education, Northeastern University, Shenyang, Liaoning, China; ^3^Department of Oncology, The Second Affiliated Hospital of Nanchang University, Nanchang, Jiangxi, China

**Keywords:** data augmentation strategies, deep learning models, lesion segmentation, pseudolabeled sample generation, ultrasound imaging

## Abstract

Automated lesion segmentation in ultrasound (US) images based on deep learning (DL) approaches plays a crucial role in disease diagnosis and treatment. However, the successful implementation of these approaches is conditioned by large-scale and diverse annotated datasets whose obtention is tedious and expertise demanding. Although methods like generative adversarial networks (GANs) can help address sample scarcity, they are often associated with complex training processes and high computational demands, which can limit their practicality and feasibility, especially in resource-constrained scenarios. Therefore, this study is aimed at exploring new solutions to address the challenge of limited annotated samples in automated lesion delineation in US images. Specifically, we propose five distinct mixed sample augmentation strategies and assess their effectiveness using four deep segmentation models for the delineation of two lesion types: breast and thyroid lesions. Extensive experimental analyses indicate that the effectiveness of these augmentation strategies is strongly influenced by both the lesion type and the model architecture. When appropriately selected, these strategies result in substantial performance improvements, with the Dice and Jaccard indices increasing by up to 37.95% and 36.32% for breast lesions and 14.59% and 13.01% for thyroid lesions, respectively. These improvements highlight the potential of the proposed strategies as a reliable solution to address data scarcity in automated lesion segmentation tasks. Furthermore, the study emphasizes the critical importance of carefully selecting data augmentation approaches, offering valuable insights into how their strategic application can significantly enhance the performance of DL models.

## 1. Introduction

Lesion delineation in ultrasound (US) images is a critical step in diagnosing, prognosing, and treating various medical conditions, including cancers. This process involves identifying lesions and distinguishing them from surrounding tissues. However, achieving accurate delineation is challenging and can be hindered by multiple factors. First, unlike other medical imaging modalities, US images often have low contrast and inconsistent intensity, as well as artifacts like speckle noise, shadowing, and reverberation, all of which obscure lesion boundaries and reduce image quality [1, 2]. Second, lesions in US images can vary significantly in shape, size, and intensity, with many exhibiting ambiguous boundaries. This variability complicates segmentation, making it a tedious and time-consuming task. Third, radiologists often face demanding workloads, which can impact delineation accuracy and lead to variability between examiners. Therefore, automating the process of lesion delineation in US images has attracted considerable attention from diverse research communities.

In recent decades, the successes of artificial intelligence (AI) in diverse application domains have led to their expansion to multiple medical US tasks, including lesion delineation. Numerous advanced approaches based on deep learning (DL) techniques have been developed for automatically segmenting lesions in US image data from different organs. Commonly studied lesion types include those found in the breast [3–5], thyroid [6, 7], and liver [8, 9]. Although these approaches have demonstrated promising performance in enhancing the accuracy, efficiency, and reproducibility of lesion delineation in US images, their practical impact remains limited. Only a few of these frameworks achieve the performance levels required for clinical use. A key factor hindering the effectiveness of DL-based methods is the lack of large-scale, diverse datasets for model training. This shortage of comprehensive data limits the models' ability to generalize effectively across different patient demographics and imaging conditions, posing a significant challenge for their clinical adoption [10].

To mitigate the challenge of data scarcity, researchers have explored diverse strategies to enhance the performance of DL frameworks for US image segmentation. These approaches include data augmentation techniques [11–13], transfer learning [14–17], semisupervised learning [18, 19], and the use of synthetic data generated through generative models [20, 21]. Other researchers specifically focused on proposing strategies to overcome data scarcity in automated lesion delineation in US images. Zhang et al. proposed a sample size enhancement approach that leverages image cutting and mirroring, followed by boundary reconstruction, to generate new annotated images [22]. The training of the DL model using the augmented dataset based on the newly generated samples resulted in a significant improvement in segmentation accuracy. Extensive experiments in breast tumor delineation in US images demonstrated superior performance, highlighting the feasibility of the proposed approach. You et al. contributed to enhancing the segmentation performance of DL frameworks for breast lesion delineation in two main aspects [23]. First, they leveraged the advantages of the image-to-image translation capability of GAN (generative adversarial network) models to produce new annotated samples from lesion target images, thereby significantly enhancing the size of the training set. Subsequently, they designed an improved DL model that combines the advantages of ResNet18, deep supervision techniques, and channel attention mechanisms to reinforce the model's learning capabilities, leading to better segmentation results. Similarly, Luo et al. employed GAN to generate new annotated samples for breast lesion analysis [24]. Their data generation process was implemented in two steps: a mask generation step aimed at creating tumor contours and an image generation step consisting of producing new breast ultrasound images (BUSIs). Experimental analyses indicated that the proposed framework outperformed conventional sample augmentation techniques, achieving significantly enhanced lesion differentiation accuracy and lesion delineation performance. In another study, Pan et al. circumvented the shortage of annotated samples by integrating an attention decoupling module and contrastive learning strategy into their segmentation framework [25]. They leveraged a copy–paste technique to produce pseudolabeled samples involving both a limited number of manually labeled samples and unlabeled samples. In addition, recent work has explored more advanced augmentation strategies. Sun et al. proposed a super-pixel-based method (LCAMix) that mixes image regions while preserving structural details, which improves segmentation accuracy but can introduce unnatural boundary transitions, particularly problematic for US images with inherently blurry edges [26]. Zhang et al. introduced a text-guided diffusion model that generates high-quality synthetic images using textual and edge-based conditions, yielding strong performance gains. However, its high computational cost and reliance on expert tuning limit its practical applicability [27].

Despite these advances, two critical limitations persist: (1) the high cost and specialized expertise required for generating synthetic annotated data and (2) the lack of comparative evaluations across different network architectures under a unified augmentation strategy. Although multiple techniques have been proposed to enhance DL-based lesion delineation, synthetic data generation remains one of the most promising approaches. Some researchers have explored GAN-based sample generation to address data scarcity [23, 24], but these methods often require complex training procedures, substantial computational resources, and expert tuning. Others, such as Zhang et al. [22] and Pan et al. [25], employed classical image processing techniques, yet did not fully leverage mixed sample generation, and comprehensive evaluations of augmentation performance across different DL models remain limited.

In response to the aforementioned unresolved issues, our study is aimed at leveraging mixed sample augmentation techniques to expand both the size and diversity of training samples, with the overall goal of improving automated lesion delineation in US images. Specifically, we design and implement five different mixed sample augmentation strategies to enable the use of unlabeled samples for network training. It is important to note that, unlike other image generation methods such as GANs, the mixed sample augmentation strategy provides a more efficient alternative by simplifying the implementation process and reducing the need for complex, resource-intensive model training. These techniques are then applied to enhance the segmentation performance of DL models for two lesion types: breast and thyroid. Furthermore, we conduct comprehensive analyses to evaluate the effectiveness of these augmentation strategies on both lesion type delineation and DL framework performance, providing valuable insights into optimizing data augmentation for more accurate segmentation outcomes. In a nutshell, our study's key contributions can be summarized as follows:

• The study introduces and implements five distinct mixed sample augmentation techniques to enhance the size and diversity of training sets, enabling the use of unlabeled samples in network training.
• The proposed mixed sample augmentation techniques are applied to improve segmentation performance in US images of breast and thyroid lesions, addressing the challenge of limited labeled data. A comprehensive evaluation across multiple lesion types and DL architectures provides actionable insights for optimizing data augmentation strategies in clinical applications.

## 2. Methodology

It is undeniable that there have been noticeable advances in DL-based lesion delineation in US images. However, the practical significance of these approaches remains limited due to relatively poor generalization capability due to the scarcity of large-scale annotated datasets for network training. Generating accurately annotated US images is a labor-intensive and time-consuming task and requires expert knowledge. Data augmentation has been proven to be the most straightforward alternative to tackle such challenges. Data augmentation approaches can be categorized into two groups: conventional approaches and advanced approaches. The former group refers to simple operations including rotation, flipping, and elastic transformation, whereas advanced approaches are those involving one or more well-known image analysis algorithms including GAN-based approaches and mixed sample approaches.

Our study is aimed at tackling the challenge of limited annotated data by introducing strategies to expand the size and diversity of training sets, a critical step in advancing automated lesion delineation in US images. Specifically, the mixed sample concept is employed to design five strategies for generating pseudolabeled samples, leveraging a small number of manual annotations alongside a large pool of unlabeled data. These mixed sample augmentation techniques are straightforward and efficient, enabling the effective use of unlabeled data while minimizing dependence on resource-intensive and complex model training processes. Through comprehensive analyses, we evaluate the effectiveness of these techniques in improving DL framework performance for delineating two lesion types, providing valuable insights into optimizing data augmentation to achieve enhanced segmentation accuracy.

### 2.1. Proposed Mixed Sample Augmentation Strategies

Mixed sample augmentation offers a computationally efficient alternative to GANs by combining existing labeled and unlabeled data through linear interpolations or geometric transformations [28]. This approach avoids the instability of adversarial training while maintaining diversity in augmented samples [29], making it particularly suitable for medical imaging tasks with limited annotated data. Building on these foundations, our study investigates the potential of mixed sample augmentation for lesion delineation in US images. Our proposed strategies are grounded in pixel-level variations, effectively simulating the domain shifts commonly encountered in real-world US imaging scenarios. Unlike confidence-based filtering approaches, our proposed techniques do not rely on model prediction scores, thereby avoiding potential bias or instability introduced by uncertain predictions. Moreover, these techniques produce pseudolabeled samples from limited annotated samples combined with large-scale unlabeled data, ensuring the generation of images that maintain authentic features without deformation or hallucination. The details of these augmentation strategies are as follows.

**Figure 1:**
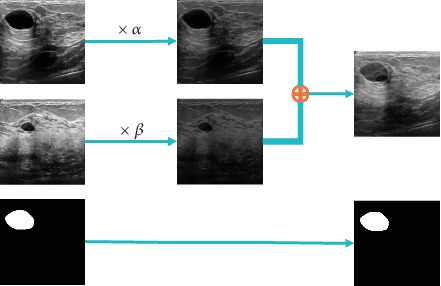
The sample generation process of PixMix.

*PixMix*: This approach is inspired by the Mixup method [30] but differs in its key focus: rather than randomly blending images, it modifies the features of the original image by selectively incorporating controlled features from another image. Importantly, the segmentation target remains unaltered, avoiding the ambiguity and unnatural artifacts that can arise with Mixup. By maintaining the integrity of the segmentation labels, this method ensures the augmented data is both realistic and reliable for model training. An overview of the sample generation process is provided in Figure 1, and the augmented sample is generated as follows:

(1) $$\begin{array}{c} \left(I_{\text{aug}},T_{\text{aug}}\right)=\left\{\begin{array}{c} I_{\text{aug}}=\alpha I_{L}+\beta I_{U},\\ T_{\text{aug}}=T_{L}, \end{array}\right. \end{array}$$

where *α* corresponds to the ratio of features kept unchanged in the original image and *β* is the ratio of features extracted from the unlabeled image and added to the original labeled image. These parameters play a key role in balancing structural fidelity with contextual enrichment. Their values were empirically determined through a sparse parameter space evaluation, as described in Section 4.6. The optimal configuration was found to be *α* = 0.8 and *β* = 0.2.

*SegMix2*: This augmentation strategy is inspired by our previous work, SegMix [17], with differences in evaluation domains and usage context. It creates new annotated samples by blending the lesion area from a labeled image, which is extracted using a manually generated lesion mask, onto an unlabeled image. This process effectively transfers the labeled lesion region onto the unlabeled image, thereby generating pseudolabeled samples. By blending these regions, the strategy enhances the diversity of the training set while maintaining accurate segmentation targets, offering a practical solution for leveraging unlabeled data in the training of DL models. The data generation process can be mathematically formulated as follows:

(2) $$\begin{array}{c} \left(I_{\text{aug}},T_{\text{aug}}\right)=\left\{\begin{array}{c} I_{\text{aug}}=\left(1-T_{L}^{\prime }\right)⊙I_{U}+T_{L}^{\prime }⊙I_{L}^{\prime },\\ T_{\text{aug}}=T_{L}, \end{array}\right.\\ I_{L}^{\prime }=\gamma I_{L}+\left(1-\gamma \right)I_{U}, \end{array}$$

where *γ* is a weighting factor to control the amount of image features exchanged between the extracted area of the labeled image and the target area within the unlabeled image.

*LesionBlend*: This approach draws inspiration from LesionMix [31] but incorporates significant modifications. In our method, the lesion area is first refined using Gaussian smoothing, which ensures a more natural transition between the blended regions. This smoothed lesion mask is then used to extract an area of that size from an unlabeled image, which is subsequently blended onto the labeled image. The background of the labeled image remains intact, while the foreground region corresponding to the lesion area is selectively replaced with new content. Moreover, unlike LesionMix, our approach does not apply spatial transformations to the lesion area after extraction. This ensures the preservation of the lesion's anatomical structure and spatial consistency, making the augmented samples more realistic and reliable for training DL models.

(3) $$\begin{array}{c} \left(I_{\text{aug}},T_{\text{aug}}\right)=\left\{\begin{array}{c} I_{\text{aug}}=T_{L}^{\prime }⊙I_{L}+\left(1-T_{L}^{\prime }\right)⊙I_{U},\\ T_{\text{aug}}=T_{L}. \end{array}\right. \end{array}$$

*LesionBlend2*: This approach builds upon the concept of LesionBlend but introduces a key distinction in its execution. Unlike LesionBlend, where the original background of the labeled image remains unchanged, this method actively modifies the background image while replacing the lesion area (foreground region) with new content. The process begins with refining the lesion mask using Gaussian smoothing, which serves as a precise guide to extract the lesion region from the labeled image. The extracted lesion is then carefully blended onto an unlabeled image, effectively embedding the lesion into a different context.

(4) $$\begin{array}{c} \left(I_{\text{aug}},T_{\text{aug}}\right)=\left\{\begin{array}{c} I_{\text{aug}}=\left(1-T_{L}^{\prime }\right)⊙I_{L}+T_{L}^{\prime }⊙I_{U},\\ T_{\text{aug}}=T_{L}. \end{array}\right. \end{array}$$

*PixMix2*: This approach is inspired by the principle of PixMix but introduces a key difference in methodology. Unlike PixMix, which incorporates controlled features of an unlabeled image into a labeled image, this method creates a new image by fully combining features from both. Specifically, the two images are directly added together to generate the final composite image, resulting in a seamless integration of their features. The augmented annotated sample is obtained as follows:

(5) $$\begin{array}{c} \left(I_{\text{aug}},T_{\text{aug}}\right)=\left\{\begin{array}{c} I_{\text{aug}}=I_{L}+I_{U},\\ T_{\text{aug}}=T_{L}. \end{array}\right. \end{array}$$

In the above equations, *I*_aug_ and *T*_aug_ correspond to the generated image and its corresponding lesion mask, respectively. *I*_*L*_ and *T*_*L*_ represent an image from the small-size annotated dataset and the corresponding manually generated lesion mask, respectively. *I*_*U*_ is an image from the unlabeled sample set. *T*_*L*_′ is the smoothed version of *T*_*L*_. The ⊙ means dot product.

**Figure 2:**
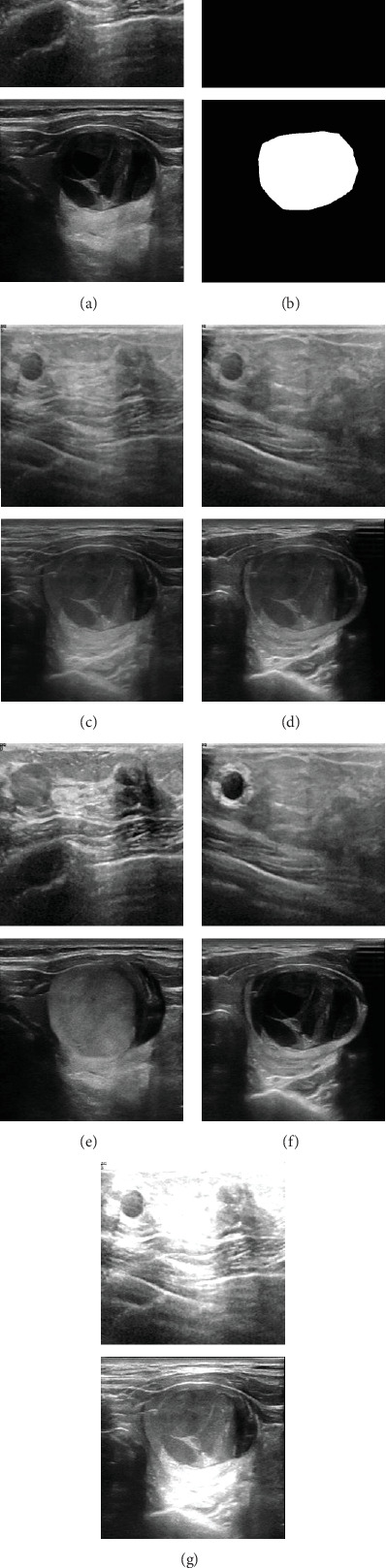
Visual comparison between the original images and the augmented samples. The first row presents breast ultrasound images, and the second row presents thyroid ultrasound images. (a, b) The original image and its corresponding lesion mask, respectively. (c–g) The augmented results generated by the PixMix, SegMix2, LesionBlend, LesionBlend2, and PixMix2 methods, respectively.

To provide an intuitive understanding of the impact of different augmentation strategies, Figure 2 presents a visual comparison between the original image and its transformed versions generated by the proposed approaches.

### 2.2. Network Models and Validation Approach

To evaluate the effectiveness of the proposed data augmentation technique in improving the performance of DL models for lesion delineation, four distinct deep segmentation models are considered: UNet [32], HEAT [33], TransUNet [34], and AttUNet [35]. The skip connections of UNet preserve low-level details but lack global context. AttUNet incorporates an attention mechanism to focus on key regions yet still fails to capture long-range dependencies. TransUNet integrates Transformer self-attention modules to enable global feature modeling. The HEAT network employs a U-shaped architecture combining convolutional and Transformer elements with coordinate residual blocks and enhanced channel self-attention to fuse local and global information, thereby improving lesion detection in complex backgrounds.

In the phase of effectiveness analysis, a series of experiments are conducted using training sets of varying sizes, which are sampled from the initially available manually annotated dataset. Specifically, three subsets comprising 5%, 10%, and 20% of the manually annotated dataset are created. For each subset, the proposed data augmentation strategies are applied to generate additional pseudolabeled samples by leveraging the limited labeled data along with a larger pool of unlabeled data. This process expands each subset to match the size of the original training set, resulting in more diverse and representative datasets. Deep segmentation models are then trained separately on (1) the limited labeled data and (2) the corresponding augmented datasets that combine labeled and pseudolabeled samples. Model performance is evaluated on the test set to assess the impact of incorporating the augmented pseudolabeled data.

### 2.3. Performance Assessment Criteria

The segmentation performance of the implemented DL models is evaluated using three commonly used metrics: the Jaccard index (JI) [36], the Dice coefficient (Dice) [37], and the Hausdorff distance (HD95) [38]. The JI measures the overlap between the predicted segmentation result and the true label. The Dice coefficient is defined as twice the intersection of the predicted and true labels, divided by the sum of their pixels. HD95 is primarily used to assess the degree of boundary overlap, with a smaller value indicating better boundary alignment. The formulas for these metrics are provided below.

(6) $$\begin{array}{c} \text{JI}=\frac{\text{TP}}{\text{TP}+\text{FP}+\text{FN}}, \end{array}$$

(7) $$\begin{array}{c} \text{Dice}=\frac{2\text{TP}}{2\text{TP}+\text{FP}+\text{FN}}, \end{array}$$

(8) $$\begin{array}{c} \text{HD}95\left(A,B\right)=\max\left\{\underset{a\in A}{\max}\underset{b\in B}{\min}d\left(a,b\right),\underset{b\in B}{\max}\underset{a\in A}{\min}d\left(a,b\right)\right\}, \end{array}$$

where TP, FP, TN, and FN represent true positives, false positives, true negatives, and false negatives, respectively. *A* and *B* represent the point sets of the predicted boundary and the ground truth boundary, respectively. *d*(*a*, *b*) denotes the distance between points *a* and *b*.

## 3. Datasets and Experimental Settings

### 3.1. Datasets

To validate the effectiveness of the proposed image generation methods, this study used two datasets: the publicly available BUSI dataset and an in-house thyroid cancer dataset. The BUSI dataset consists of 780 BUSIs, including 437 benign cases, 210 malignant cases, and 133 normal cases. All images were obtained using the LOGIQ E9 and LOGIQ E9 Agile US systems, and the ground truth annotations were provided by radiologists from Baheya Hospital. For our experimental dataset, we excluded the images of normal cases and selected 647 benign and malignant cases to construct the dataset. The training set consists of 414 cases, the validation set includes 103 cases, and the test set contains 130 cases. The in-house thyroid cancer dataset was collected from the Second Affiliated Hospital of Nanchang University using the Mindray Resona 7s device. The dataset consists of 82 cases, divided into 72 for training and 10 for testing. For each case, an average of 32 frames were extracted from the patient videos, resulting in a total of 2300 images in the training set and 300 images in the test set. Furthermore, the training set was further split into 80% (1840 images) for training and 20% (460 images) for validation. The thyroid lesions were initially annotated by a radiologist with 5 years of clinical experience, followed by a review by a senior radiologist with 15 years of experience to ensure the accuracy of the final annotations.

### 3.2. Experimental Settings

The DL models considered in this study were implemented using the PyTorch framework. All training and testing processes were carried out on a GeForce RTX 2080Ti GPU with 11GB of memory, using PyTorch 1.8.1, Python 3.8, and CUDA 11.1. The models were optimized using stochastic gradient descent (SGD), with an initial learning rate set to 0.01 and a batch size of 16. Through empirical analyses, SoftDiceLoss was chosen as the loss function. During training, all images were resized to 224 × 224, and random horizontal flipping with a probability of 0.5 was applied.

## 4. Results and Discussions

The primary objective of our study is to investigate the impact of various data augmentation strategies on the segmentation performance of DL models for automated lesion delineation in US images. Given the complexity and variability of lesions, it is crucial to assess how these augmentation techniques affect the model's ability to accurately segment different types of lesions. Our study is aimed not only at evaluating the overall impact of data augmentation on segmentation accuracy but also at determining the most effective augmentation approach for specific lesion types. Identifying appropriate data augmentation strategies is crucial, especially when working with a limited number of manually annotated samples for network training. Moreover, while the optimal data augmentation strategy may vary depending on the lesion type, it can also be influenced by the architecture of the DL model. This underscores the importance of thoroughly assessing the model's behavior when applied to different lesion types and across various augmentation strategies, ensuring the most effective approach for each scenario.

### 4.1. Performance Assessment for Breast Lesion Delineation

**Figure 3:**
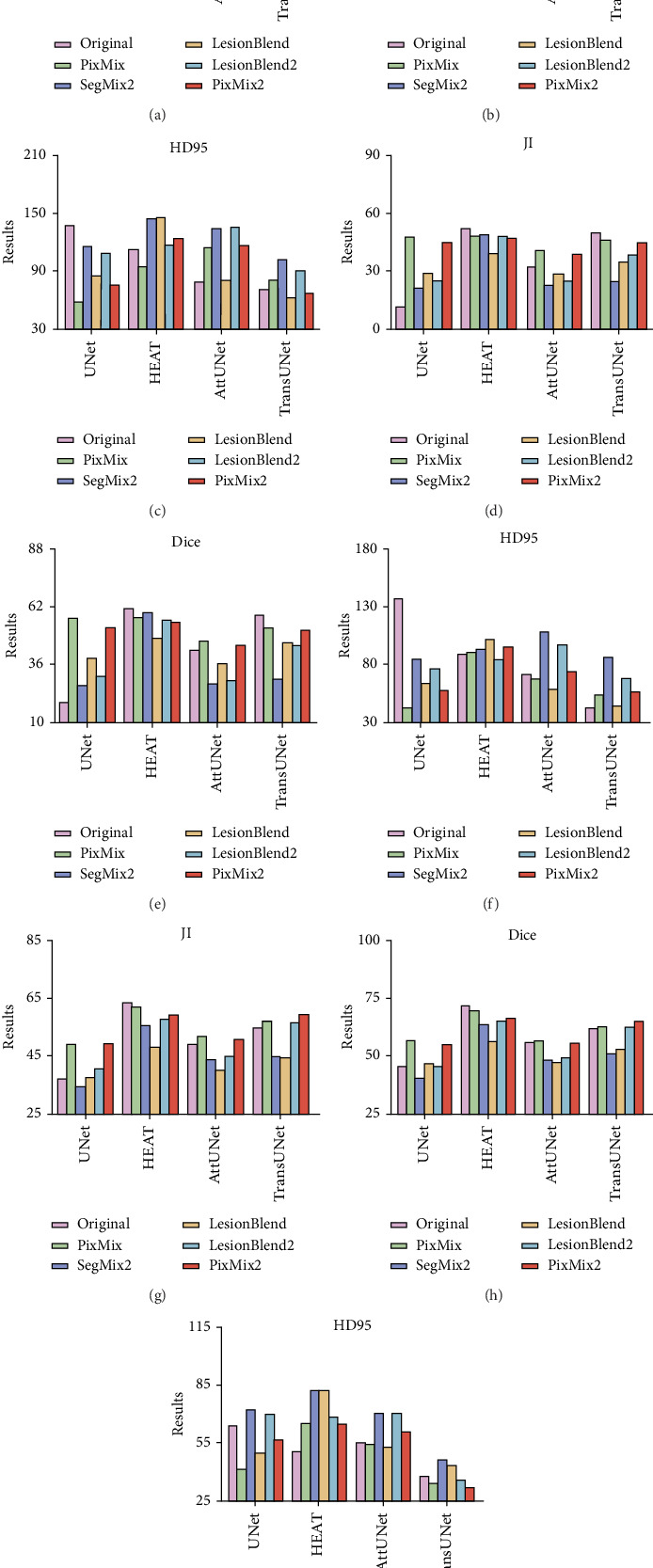
Quantitative evaluation of segmentation performance on the BUSI dataset. The comparison of the results of applying five pseudolabeled image generation methods under (a–c) 5%, (d–f) 10%, and (g–i) 20% data. For each model, the results from left to right correspond to original (pink), PixMix (green), SegMix2 (purple), LesionBlend (orange), LesionBlend2 (blue), and PixMix2 (red).

Figure 3 presents a comparison of the segmentation results from the five pseudolabeled image generation methods we proposed, applied to varying proportions of data from the BUSI dataset. The first, second, and third rows correspond to the segmentation performance using 5%, 10%, and 20% of the available manually annotated samples, respectively, combined with a larger pool of pseudolabeled samples generated through our proposed augmentation strategies. It can be observed that the performance of the DL models varies with different data percentages and augmentation strategies. Specifically, while some models exhibit improved performance under certain augmentation techniques, others show little to no enhancement.

It is found that the most suitable image augmentation strategies for breast lesion delineation are PixMix and PixMix2. The Dice coefficient and JI increased by 1.21%–37.95% and 0.8%–36.32%, respectively, while the HD95 value decreased from 13.91% to 94%. The most significant improvements are observed with the UNet model when 5% and 10% of the annotated data are used for training. Specifically, the Dice value increased by 37.95% and 24.95%, the JI value increased by 36.32% and 23.31%, and the HD95 value decreased by 94% and 78.73%, respectively. Additionally, for the other DL models, although the improvements in Dice and JI values were limited, there was a significant reduction in the HD95 value. This suggests that the designed image augmentation methods enhanced the model's ability to delineate lesion boundaries more accurately, which could help address boundary segmentation challenges, such as those caused by low contrast in US images. For example, when the LesionBlend approach was applied with 20% of the data, the Dice and JI values for the UNet network increased modestly by 1.21% and 0.8%, but the HD95 value decreased by 13.91%. This indicates that, despite the limited improvement in overlap metrics, the augmentation method significantly enhanced boundary accuracy, bringing the segmentation results closer to manual annotations and improving overall performance.

**Figure 4:**
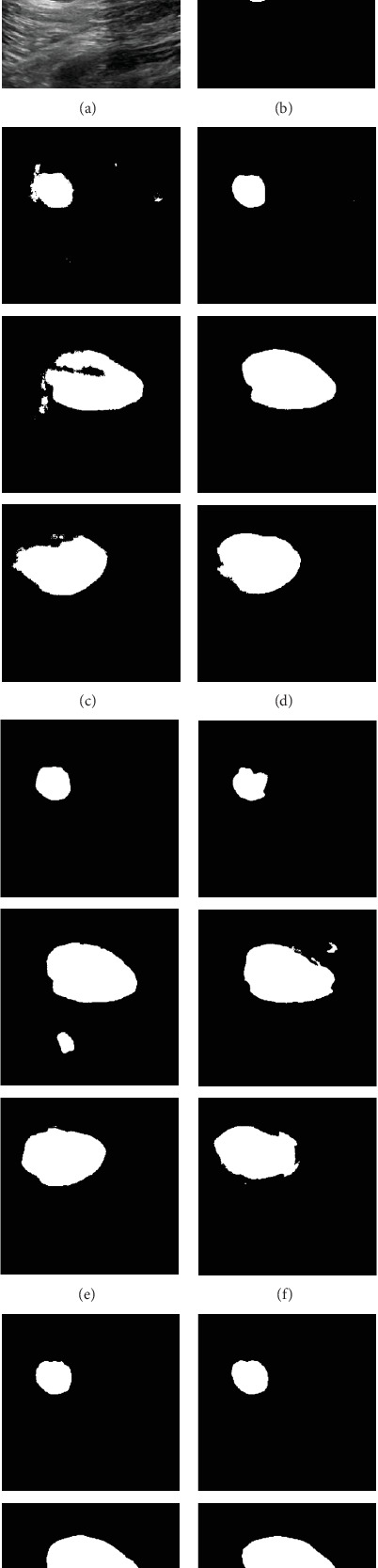
Representative segmentation results of breast ultrasound images under different augmentation strategies using the UNet model. From left to right: (a) original image, (b) ground truth, (c) segmentation result using 5% of the original annotated dataset, and (d–h) segmentation results obtained from images augmented by PixMix, SegMix2, LesionBlend, LesionBlend2, and PixMix2, respectively.

Figure 4 illustrates representative segmentation outcomes of breast lesions using the UNet model. For each case, the first two columns present the original US image and its corresponding ground truth annotation; the third column illustrates the result obtained when training with only 5% of the original labeled data. The subsequent five columns correspond to the outputs produced by our proposed augmentation methods: PixMix, SegMix2, LesionBlend, LesionBlend2, and PixMix2. Compared to the baseline performance achieved without augmentation, these methods yield notable improvements in delineating lesion boundaries, thereby underscoring the efficacy of mixed sample augmentation techniques for boundary detection.

**Figure 5:**
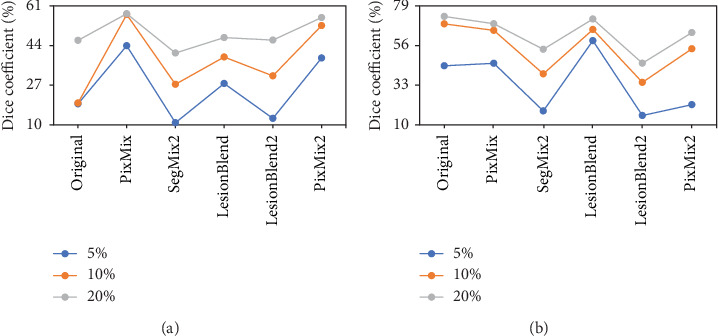
Trend of Dice coefficient for segmentation of breast ultrasound images using UNet model for the original image and different augmentation strategies. (a, b) Dice coefficient variation trend plots for breast data and thyroid data, respectively.

Figure 5a quantitatively validates these observations by illustrating the variation in Dice similarity coefficients before and after applying different augmentation strategies on the breast US dataset. As shown in the figure, the performance gains associated with each augmentation method remain consistent across varying annotation proportions, suggesting enhanced robustness and generalizability under data-scarce conditions. Notably, when only 5% of the annotated samples are used, PixMix and PixMix2 achieve the most pronounced improvements, demonstrating their practical utility in low-resource settings.

The segmentation results, as shown through quantitative measures, clearly demonstrate that for breast lesion delineation, the choice of sample generation approach to expand and diversify the training set should be carefully tailored to the specific deep network architecture. Additionally, the proposed sample augmentation strategies offer effective solutions for improving delineation performance, provided the technique is thoughtfully chosen in consideration of the model architecture.

### 4.2. Performance Assessment for Thyroid Lesion Delineation

**Figure 6:**
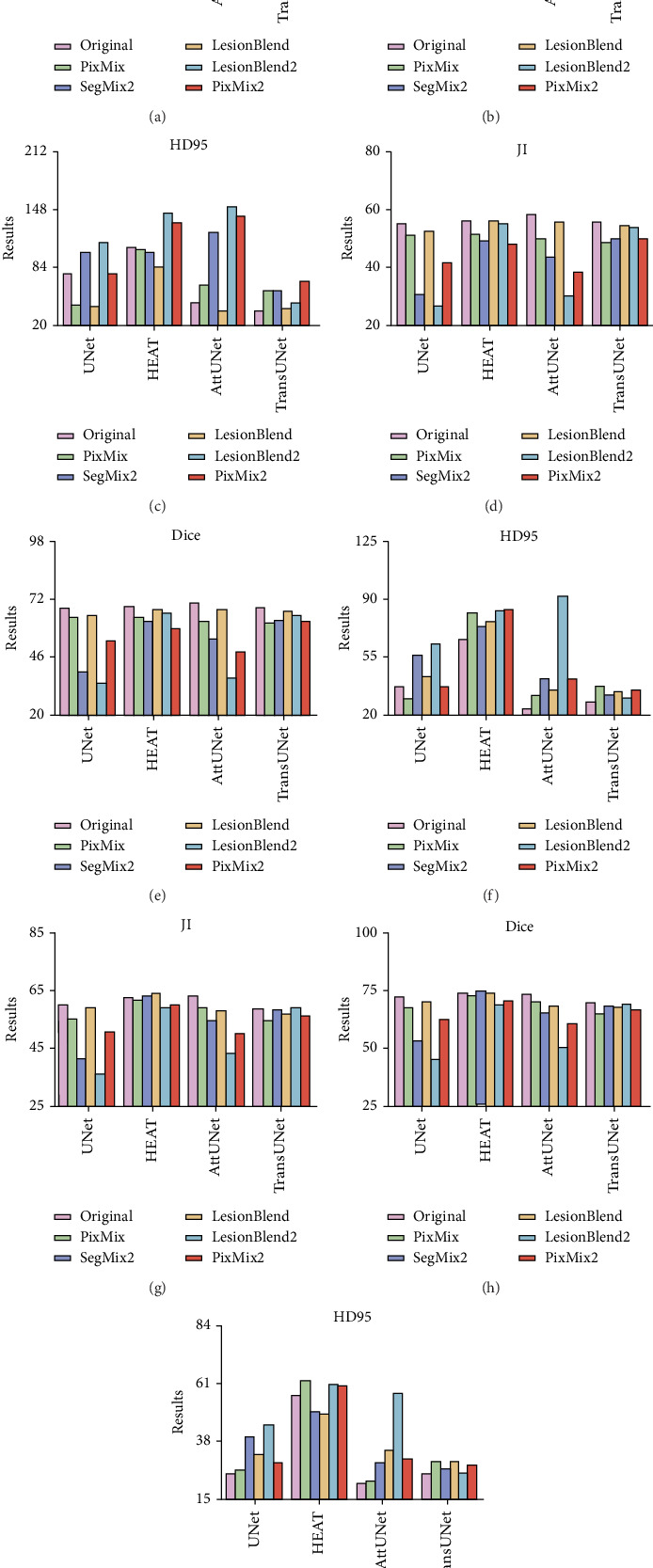
Quantitative evaluation of segmentation performance on in-house dataset. The comparison of the results of applying five pseudolabeled image generation methods under (a–c) 5%, (d–f) 10%, and (g–i) 20% data. For each model, the results from left to right correspond to original (pink), PixMix (green), SegMix2 (purple), LesionBlend (orange), LesionBlend2 (blue), and PixMix2 (red).

Figure 6 quantitatively evaluates the effectiveness of the five pseudolabeled image generation strategies applied to our in-house thyroid nodule dataset at different sample proportions. The first, second, and third rows depict the segmentation performance achieved with 5%, 10%, and 20% of manually labeled samples, respectively, combined with the application of the proposed augmentation strategies. As observed, with the increase in the percentage of data used, the effectiveness of the augmentation strategies tends to diminish. In some cases, certain models even showed a decline in segmentation performance after applying specific augmentation methods.

**Figure 7:**
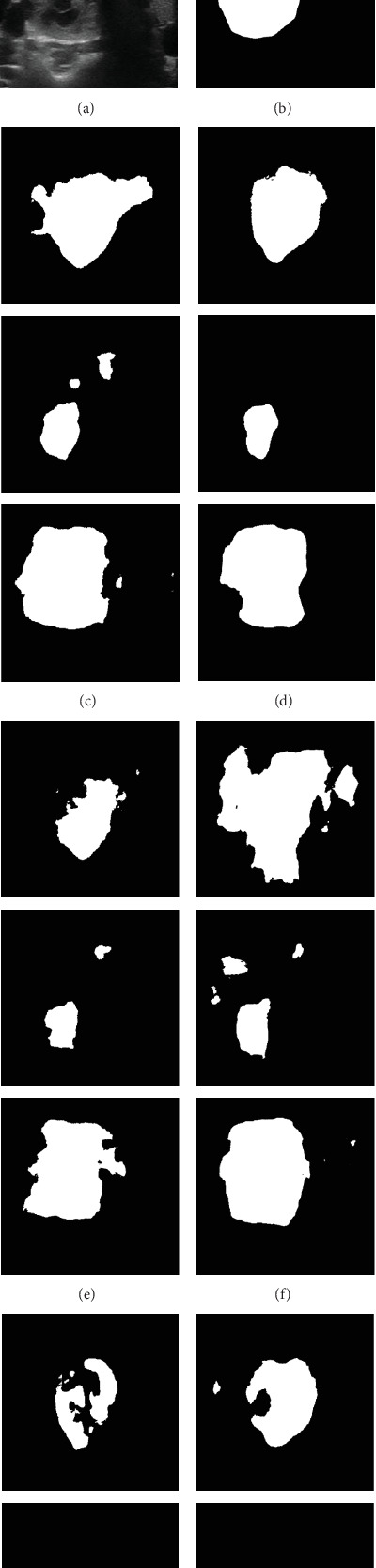
Representative segmentation results of thyroid ultrasound images under different augmentation strategies. From left to right: (a) original image, (b) ground truth, (c) segmentation result using 5% of the original annotated dataset, and (d–h) segmentation results obtained from images augmented by PixMix, SegMix2, LesionBlend, LesionBlend2, and PixMix2, respectively.

In the context of thyroid lesion delineation, the LesionBlend and PixMix methods were found to be the most effective data augmentation strategies, significantly enhancing segmentation performance. The increases in JI and Dice values ranged from 1.92% to 13.01% and 2.68% to 14.59%, while the decreases in HD95 values ranged from 7.42% to 36.71%. Specifically, the UNet and HEAT networks showed the greatest improvements with 5% samples, with the Dice values increasing by 14.59% and 7.6%, JI values increasing by 13.01% and 9.44%, and HD95 values decreasing by 36.71% and 22.06%, respectively. To further substantiate these quantitative results, Figure 7 provides representative visualizations of segmentation outcomes under different augmentation strategies using the UNet model. The qualitative comparisons reveal perceptible improvements in lesion delineation, especially in cases involving limited training data. Moreover, for 10% and 20% of the initially available annotated training set, the data augmentation strategies did not yield satisfactory improvements. This could be due to the sufficient size of the initial dataset at these percentages, which may have already provided enough variability for the model, thereby limiting the added benefit of augmentation techniques. As shown in Figure 5b, the performance gains of augmentation strategies such as LesionBlend and PixMix gradually diminish as the proportion of annotated data increases. This trend suggests that when sufficient training data is available, the model can already learn lesion features effectively, making the additional diversity introduced by augmentation less impactful. It is important to note that if the augmentation strategies generate samples whose distribution significantly deviates from that of the original dataset, this can lead to underperformance rather than improvement.

### 4.3. Tailoring Augmentation Strategies to DL Models for Lesion Segmentation

This study highlights the importance of tailoring data augmentation strategies to both the lesion type and the specific architecture of DL models for optimal lesion segmentation performance. For the breast lesion delineation, the UNet, HEAT, and AttUNet models exhibited the best segmentation improvements after applying the PixMix and PixMix2 methods. Notably, the UNet model achieved the most substantial improvement, demonstrating more than four times the performance increase compared to AttUNet. In contrast, other augmentation strategies, such as LesionBlend, led to a performance drop when applied to these networks. Additionally, the TransUNet model performed best with the PixMix2 method, while it underperformed with SegMix2, LesionBlend, and LesionBlend2. For the thyroid lesion delineation task, the most effective data augmentation strategies were LesionBlend and PixMix when applied to the UNet and HEAT models. However, these models performed poorly when using LesionBlend2 and SegMix2. In contrast, the TransUNet model exhibited superior performance with the LesionBlend2 method.

These findings indicate that while some data augmentation techniques are effective across multiple models, others may require more tailored approaches based on the model's architecture. Moreover, even for the same lesion type, the optimal augmentation technique can vary depending on the network architecture. Therefore, selecting appropriate data augmentation methods should consider both the lesion type and the underlying architecture of the DL model. This highlights that image augmentation methods should not be generalized across models or lesion types, as their effectiveness can vary significantly depending on these factors.

### 4.4. Optimizing Augmentation Strategies for Lesion Segmentation: A Type-Specific Approach With a Consistent Model

The experimental results indicate that for the delineation of different lesion types, the optimal data augmentation strategy may vary even when applied to the same DL model. For example, for breast lesion delineation, applying PixMix to the UNet, HEAT, and AttUNet models showed the most remarkable performance improvements. In contrast, the LesionBlend method yielded the best results for thyroid lesion segmentation with these models. Moreover, for the TransUNet model, the optimal performance on breast lesion was achieved with the PixMix2 technique, while the most suitable augmentation strategy for thyroid lesion was LesionBlend2. These results highlight that even for the same network model, the most effective augmentation strategy can differ based on the type of lesion being segmented. Therefore, the choice of augmentation strategies for lesion segmentation requires not only considering the type of lesion but also the inherent strengths and weaknesses of the network architecture. The integration of augmentation strategies should be viewed as part of a broader model optimization process that involves both architectural and dataset characteristics to achieve the best performance.

### 4.5. Comparative Evaluation With Traditional Augmentation Methods

To further validate the effectiveness of the proposed mixed sample augmentation strategies, we conducted additional comparative experiments using three widely adopted traditional augmentation techniques: rotation, translation, and Gaussian noise. These methods are commonly considered fundamental and practical tools for enhancing medical image segmentation datasets. Kozah et al. highlighted that geometric transformations like rotation and translation improve robustness to spatial variations, while Gaussian noise enhances generalization to varying image quality [39]. Furthermore, Goceri emphasized that combining these basic techniques effectively boosts training diversity and model performance in low-data scenarios [40].

In our comparative experiments, we used 5% of the original training set for both breast and thyroid lesion segmentation tasks. Each of the three traditional augmentation techniques was applied independently to expand this subset to match the size of the full original training set. For rotation, image-label pairs were randomly rotated by angles between 15° and 345°, deliberately avoiding minor adjustments near 0° or 360° to ensure substantial geometric variation. Translation involved uniformly shifting images and labels by ±20% along both horizontal and vertical axes, simulating spatial displacement due to probe movement or patient repositioning. Gaussian noise was added to the images (leaving the labels unchanged) using zero-mean noise with a variance range of 10–50, introducing realistic texture perturbations without degrading lesion boundary visibility. These settings were chosen to simulate common clinical variations and enhance the model's robustness to spatial and intensity-based changes.

The segmentation results obtained using the three traditional augmentation techniques, along with those achieved by the proposed mixed sample augmentation strategies, are presented in Table 1. As shown, the mixed sample augmentation approaches outperform the traditional methods, demonstrating their superior effectiveness in enhancing model performance. Specifically, on the breast dataset, PixMix achieved the most significant performance gains over traditional methods when applied to the UNet model, with JI and Dice improving by 23.31% and 24.95%, respectively, and HD95 decreasing by 78.79%. While the HEAT model did not show notable improvements in JI and Dice after applying PixMix, it exhibited a reduction in HD95, suggesting enhanced boundary delineation. For the thyroid dataset, LesionBlend emerged as the most effective strategy, particularly for the UNet model, which achieved JI and Dice improvements of 13.01% and 14.59%, respectively, along with a 36.71% reduction in HD95. These findings demonstrate that the proposed mixed sample augmentation strategies can substantially improve segmentation accuracy and boundary precision, especially under limited data conditions.

### 4.6. Ablation Study on the Weighting Parameters *α* and *β* in the PixMix Approach

To determine appropriate values for *α* and *β*, we conducted a sparse parameter sweep to assess their impact on model performance. Specifically, we randomly selected 5% of the labeled samples from the BUSI dataset and applied the PixMix strategy to generate pseudolabeled samples using different parameter settings, with *α* ∈ {0.6, 0.7, 0.8} and *β* = 1 − *α*. Subsequently, the four implemented segmentation models were trained using the augmented datasets generated under each parameter combination. The resulting segmentation performances are summarized in Table 2, evaluated in terms of Dice, JI, and HD. It can be observed that most models achieve their best performance with the parameter setting *α* = 0.8 and *β* = 0.2. Among them, AttUNet demonstrates the most notable improvement, with its JI and Dice scores increasing by 7.45% and 9.13%, respectively, compared to the setting *α* = 0.6 and *β* = 0.4. Similarly, TransUNet exhibits an 18.02% reduction in HD95 under the *α* = 0.8 and *β* = 0.2 configuration. Although UNet achieves slightly higher JI and Dice scores at *α* = 0.7 and *β* = 0.3, both HEAT and TransUNet report HD95 reductions of 21.57% and 6.27%, respectively, under *α* = 0.8 and *β* = 0.2. Based on these results, *α* = 0.8 and *β* = 0.2 are selected as the optimal configuration for the PixMix strategy, offering the most effective and stable performance across different segmentation models.

### 4.7. Limitations and Future Works

Despite the promising performance improvements observed with the proposed data augmentation strategies and the valuable insights gained into their adoption, our study presents several limitations that require further investigation.

First, while our proposed data augmentation strategies can address the challenge of limited dataset sizes and improve the model's lesion segmentation performance, the results are still insufficient to meet clinical standards. To achieve further improvements, future efforts will focus on integrating mainstream data manipulation techniques and exploring advanced optimization strategies, such as pretraining and knowledge distillation.

Second, the effectiveness of the proposed augmentation strategies was evaluated independently, even though combining multiple strategies could potentially have a more significant impact on DL model performance. Future work will focus on investigating various combinations of augmentation methods to identify synergies that can further enhance segmentation accuracy and overall model performance.

Third, this study primarily focused on two specific lesion types: breast tumors and thyroid nodules. While these datasets provided important insights, the conclusions regarding the most effective augmentation strategies may not necessarily extend to other lesion types or organs with distinct morphological and imaging features. To ensure broader applicability, future research will explore additional lesion types and segmentation targets to further validate and refine the effectiveness of the proposed augmentation strategies.

## 5. Conclusion

In this study, we propose five mixed sample augmentation strategies aimed at improving the performance of automatic lesion segmentation in US images. Unlike conventional augmentation methods such as Mixup and CutMix, which typically rely on global mixing or region-level substitution, our approach introduces a controlled, lesion-preserving augmentation strategy that generates pseudolabeled samples from unlabeled data. These augmentations are task-specific, keeping the segmentation mask invariant by selectively enhancing lesion regions. The proposed strategies are architecture-independent, computationally lightweight, and do not require additional model complexity. Our experiments on breast and thyroid US datasets demonstrate performance improvements, especially in annotation-limited scenarios, confirming the utility and robustness of the proposed approach.

In contrast to generative augmentation methods that require additional training cost and often introduce model instability, our approach achieves sample diversity through explicit pixel-level augmentation. This design ensures real-time applicability and strong parameter compliance. Furthermore, our findings suggest that blindly transplanting augmentation strategies from other domains may not be optimal; instead, a principled process of task- and architecture-aware adaptation is essential.

## Figures and Tables

**Table 1:** Quantitative comparison of five mixed sample augmentation strategies and traditional data augmentation methods.

**Dataset**	**Model**	**Metric**	**Original**	**PixMix**	**SegMix2**	**LesionBlend**	**LesionBlend2**	**PixMix2**	**Rotation**	**Translation**	**Gaussian**
BUSI	UNet	JI	11.79	**35.10**	7.81	19.59	9.63	32.09	32.35	29.27	32.17
		Dice	18.96	**43.91**	10.85	27.69	12.74	38.63	41.51	38.06	40.39
		HD95	137.57	**58.84**	116.68	86.35	109.61	76.46	65.82	78.41	68.19
	HEAT	JI	**44.95**	43.57	36.80	22.21	42.34	37.86	44.10	39.52	34.81
		Dice	54.39	52.02	46.50	31.60	49.23	45.91	**54.82**	47.66	42.62
		HD95	112.99	**95.56**	145.44	146.41	117.83	124.33	98.91	122.56	115.45
	TransUNet	JI	36.40	29.34	15.35	23.90	17.81	34.59	**44.67**	38.47	38.88
		Dice	48.84	34.51	19.63	33.63	22.76	40.99	**53.19**	46.58	48.63
		HD95	70.85	82.15	102.08	63.07	91.15	68.65	**51.26**	67.73	54.75
	AttUNet	JI	18.82	14.80	7.96	15.83	9.00	15.10	**30.10**	29.92	28.19
		Dice	25.17	18.35	9.92	21.40	10.83	17.63	**37.96**	36.00	37.17
		HD95	**79.31**	114.93	134.87	81.71	136.38	117.42	82.45	83.34	81.84

In house	UNet	JI	31.43	33.32	13.73	**44.44**	12.16	16.17	33.52	40.98	39.95
		Dice	44.23	45.66	18.00	**58.82**	15.34	21.66	45.78	53.86	53.82
		HD95	78.97	44.00	102.79	**42.26**	114.86	79.37	46.21	39.91	42.92
	HEAT	JI	40.02	44.02	41.68	**49.46**	34.75	33.03	38.24	44.06	44.22
		Dice	54.16	55.84	55.86	**61.73**	44.18	43.18	51.42	57.89	57.78
		HD95	108.90	106.61	103.25	**86.84**	147.29	136.91	109.65	90.54	95.42
	TransUNet	JI	43.14	26.63	25.08	**45.06**	36.22	22.74	37.21	44.92	41.27
		Dice	56.52	36.14	35.01	**59.20**	47.15	30.75	50.12	57.60	53.65
		HD95	37.42	60.68	60.73	**40.07**	47.47	70.87	44.03	34.83	40.45
	AttUNet	JI	42.26	25.80	15.62	**47.53**	12.47	11.32	30.78	41.06	38.98
		Dice	54.55	34.10	19.27	**61.34**	14.94	13.85	40.12	53.15	50.33
		HD95	46.00	66.37	125.83	**37.27**	154.91	144.75	62.96	39.48	42.03

*Note:* The best results are highlighted in bold.

**Table 2:** Comparison of segmentation performance under varying *α* and *β* settings in the PixMix strategy.

**Model**	**Metric**	**α** = 0.8, **β** = 0.2	**α** = 0.7, **β** = 0.3	**α** = 0.6, **β** = 0.4
UNet	JI	39.77	**40.45**	38.13
	Dice	49.36	**50.32**	47.50
	HD95	62.60	58.68	**57.94**

HEAT	JI	**43.56**	42.98	43.34
	Dice	**51.97**	51.95	50.34
	HD95	**96.36**	117.93	106.97

TransUNet	JI	**45.23**	44.53	41.67
	Dice	**53.33**	52.48	48.65
	HD95	**41.05**	47.32	59.07

AttUNet	JI	**37.50**	37.14	30.05
	Dice	**44.62**	44.41	35.49
	HD95	71.36	**70.35**	71.76

*Note:* The best results are highlighted in bold.

## Data Availability

To protect patient privacy, the internal datasets and Python code used in this study are not being shared publicly at this time but are available from the authors upon reasonable request.

## References

[1] Monkam P., Lu W., Jin S. (2023). US-Net: A Lightweight Network for Simultaneous Speckle Suppression and Texture Enhancement in Ultrasound Images. *Computers in Biology and Medicine*.

[2] Li Y., Lu W., Monkam P., Wang Y. (2023). IA-Noise2Noise: An Image Alignment Strategy for Echocardiography Despeckling. *Proceedings of the 2023 IEEE International Ultrasonics Symposium (IUS)*.

[3] Wang J., Liang J., Xiao Y., Zhou J. T., Fang Z., Yang F. (2024). TaiChiNet: Negative-Positive Cross-Attention Network for Breast Lesion Segmentation in Ultrasound Images. *IEEE Journal of Biomedical and Health Informatics*.

[4] Chen G., Li L., Dai Y., Zhang J., Yap M. H. (2023). AAU-Net: An Adaptive Attention U-Net for Breast Lesions Segmentation in Ultrasound Images. *IEEE Transactions on Medical Imaging*.

[5] Ning Z., Zhong S., Feng Q., Chen W., Zhang Y. (2022). SMU-Net: Saliency-Guided Morphology-Aware U-Net for Breast Lesion Segmentation in Ultrasound Image. *IEEE Transactions on Medical Imaging*.

[6] Zheng T., Qin H., Cui Y. (2023). Segmentation of Thyroid Glands and Nodules in Ultrasound Images Using the Improved U-Net Architecture. *BMC Medical Imaging*.

[7] Li C., Du R., Luo Q., Wang R., Ding X. (2023). A Novel Model of Thyroid Nodule Segmentation for Ultrasound Images. *Ultrasound in Medicine & Biology*.

[8] Ryu H., Shin S. Y., Lee J. Y., Lee K. M., Kang H., Yi J. (2021). Joint Segmentation and Classification of Hepatic Lesions in Ultrasound Images Using Deep Learning. *European Radiology*.

[9] Fallahpoor M., Nguyen D., Montahaei E. (2024). Segmentation of Liver and Liver Lesions Using Deep Learning. *Physical and Engineering Sciences in Medicine*.

[10] Monkam P., Jin S., Lu W. (2022). An Efficient Annotated Data Generation Method for Echocardiographic Image Segmentation. *Computers in Biology and Medicine*.

[11] Smistad E., Johansen K. F., Iversen D. H., Reinertsen I. (2018). Highlighting Nerves and Blood Vessels for Ultrasound-Guided Axillary Nerve Block Procedures Using Neural Networks. *Journal of Medical Imaging*.

[12] Østvik A., Salte I. M., Smistad E. (2021). Myocardial Function Imaging in Echocardiography Using Deep Learning. *IEEE Transactions on Medical Imaging*.

[13] Monkam P., Jin S., Tang B., Zhou X., Lu W. (2022). A Disentanglement and Fusion Data Augmentation Approach for Echocardiography Segmentation. *Proceedings of the 2022 IEEE International Ultrasonics Symposium (IUS)*.

[14] Song Y., Zheng J., Lei L., Ni Z., Zhao B., Hu Y. (2022). CT2US: Cross-Modal Transfer Learning for Kidney Segmentation in Ultrasound Images With Synthesized Data. *Ultrasonics*.

[15] Behboodi B., Amiri M., Brooks R., Rivaz H. (2020). Breast Lesion Segmentation in Ultrasound Images With Limited Annotated Data. *Proceedings of the 2020 IEEE 17th International Symposium on Biomedical Imaging (ISBI)*.

[16] Amiri M., Brooks R., Rivaz H. (2020). Fine-Tuning U-Net for Ultrasound Image Segmentation: Different Layers, Different Outcomes. *IEEE Transactions on Ultrasonics, Ferroelectrics, and Frequency Control*.

[17] Monkam P., Jin S., Lu W. (2023). Annotation Cost Minimization for Ultrasound Image Segmentation Using Cross-Domain Transfer Learning. *IEEE Journal of Biomedical and Health Informatics*.

[18] Chen F., Chen L., Kong W. (2023). Deep Semi-Supervised Ultrasound Image Segmentation by Using a Shadow Aware Network With Boundary Refinement. *IEEE Transactions on Medical Imaging*.

[19] Wen M., Shcherbakov P., Xu Y. (2024). A Temporal Enhanced Semi-Supervised Training Framework for Needle Segmentation in 3D Ultrasound Images. *Physics in Medicine & Biology*.

[20] Jin S., Monkam P., Lu W. (2022). Echocardiography Segmentation Based on Cross-modal Data Augmentation Method. *Proceedings of the 2022 IEEE International Ultrasonics Symposium (IUS)*.

[21] Peng B., Huang X., Wang S., Jiang J. (2019). A Real-Time Medical Ultrasound Simulator Based on a Generative Adversarial Network Model. *Proceedings of the 2019 IEEE International Conference on Image Processing (ICIP)*.

[22] Zhang C., Bao N., Sun H. (2022). A Deep Learning Image Data Augmentation Method for Single Tumor Segmentation. *Frontiers in Oncology*.

[23] You G., Qin Y., Zhao C. (2023). A cGAN-Based Tumor Segmentation Method for Breast Ultrasound Images. *Physics in Medicine & Biology*.

[24] Luo J., Zhang H., Zhuang Y. (2023). 2S-BUSGAN: A Novel Generative Adversarial Network for Realistic Breast Ultrasound Image With Corresponding Tumor Contour Based on Small Datasets. *Sensors*.

[25] Pan P., Chen H., Li Y., Peng W., Cheng L. (2024). Attention Decoupled Contrastive Learning for Semi-Supervised Segmentation Method Based on Data Augmentation. *Physics in Medicine & Biology*.

[26] Sun D., Dornaika F., Charafeddine J. (2024). LCAMix: Local-and-Contour Aware Grid Mixing Based Data Augmentation for Medical Image Segmentation. *Information Fusion*.

[27] Zhang Z., Yao L., Wang B. (2024). DiffBoost: Enhancing Medical Image Segmentation via Text-Guided Diffusion Model. *IEEE Transactions on Medical Imaging*.

[28] Ghiasi G., Cui Y., Srinivas A. (2021). Simple Copy-Paste Is a Strong Data Augmentation Method for Instance Segmentation. *Proceedings of the IEEE/CVF Conference on Computer Vision and Pattern Recognition*.

[29] Lin Y., Wang Z., Cheng K.-T., Chen H. (2022). InsMix: Towards Realistic Generative Data Augmentation for Nuclei Instance Segmentation. *International Conference on Medical Image Computing and Computer-Assisted Intervention*.

[30] Zhang H., Cisse M., Dauphin Y., Lopez-Paz D. (2018). Mixup: Beyond Empirical Risk Management. *6th International Conference on Learning Representations (ICLR)*.

[31] Basaran B. D., Zhang W., Qiao M., Kainz B., Matthews P. M., Bai W. (2024). LesionMix: A Lesion-Level Data Augmentation Method for Medical Image Segmentation. *International Conference on Medical Image Computing and Computer-Assisted Intervention*.

[32] Ronneberger O., Fischer P., Brox T. (2015). U-Net: Convolutional Networks for Biomedical Image Segmentation. *Medical Image Computing and Computer-Assisted Intervention–MICCAI 2015: 18th International Conference*.

[33] Jiang T., Xing W., Yu M., Ta D. (2023). A Hybrid Enhanced Attention Transformer Network for Medical Ultrasound Image Segmentation. *Biomedical Signal Processing and Control*.

[34] Chen J., Lu Y., Yu Q. (2021). TransUNet: Transformers Make Strong Encoders for Medical Image Segmentation. https://arxiv.org/abs/2102.04306.

[35] Oktay O., Schlemper J., Folgoc L. L. (2018). Attention U-Net: Learning Where to Look for the Pancreas. https://arxiv.org/abs/1804.03999.

[36] Gong H., Chen J., Chen G., Li H., Li G., Chen F. (2023). Thyroid Region Prior Guided Attention for Ultrasound Segmentation of Thyroid Nodules. *Computers in Biology and Medicine*.

[37] Shamir R. R., Duchin Y., Kim J., Sapiro G., Harel N. (2019). Continuous Dice Coefficient: A Method for Evaluating Probabilistic Segmentations. https://arxiv.org/abs/1906.11031.

[38] Zhou J., Hou Z., Lu H. (2023). A Deep Supervised Transformer U-Shaped Full-Resolution Residual Network for the Segmentation of Breast Ultrasound Image. *Medical Physics*.

[39] Kozah N., Dornaika F., Charafeddine J., El Jaam J. (2024). Data Augmentation Techniques for Medical Image Segmentation – A Review. *Proceedings of the 2024 International Conference on Computer and Applications (ICCA)*.

[40] Goceri E. (2023). Medical Image Data Augmentation: Techniques, Comparisons and Interpretations. *Artificial Intelligence Review*.

